# Early Markers for Dementia in the Intellectual Disability Population: A Systematic Literature Review

**DOI:** 10.1111/jar.70144

**Published:** 2025-10-23

**Authors:** Jade Dunning, Melanie Hodgkinson, Mark Rose, Warren Dunger

**Affiliations:** ^1^ School of Psychology University of Southampton Southampton UK; ^2^ Learning Disabilities Psychology Team Dorset Healthcare University NHS Foundation Trust Poole UK

**Keywords:** ageing, dementia, early markers, intellectual disability, systematic review

## Abstract

**Background:**

Adults with intellectual disability, especially those with Down syndrome, are at increased risk of dementia. Whilst memory decline is often considered the earliest symptom, emerging research indicates decline in language, executive function, and non‐cognitive domains may also occur early, potentially before memory changes.

**Method:**

A systematic review using narrative synthesis was conducted to evaluate recent literature on early symptoms of dementia and the trajectory of decline in people with intellectual disabilities and Down syndrome.

**Results:**

Eighteen peer‐reviewed studies met the inclusion criteria. Early decline was observed across multiple domains, with memory and executive function frequently identified as sensitive to early decline. Attention, mobility, and behaviour were also commonly reported.

**Conclusion:**

The findings suggest that early dementia symptoms extend beyond memory decline, highlighting the need to broaden current assessment guidelines. Further research is required to address methodological limitations and improve early detection in this population.

**Summary**

- People with intellectual disabilities, and especially those with Down syndrome, are at higher risk of developing a condition called ‘dementia’. People with Down syndrome have an extra chromosome 21 which is thought to increase their risk of dementia. Dementia can cause changes in memory and other skills, but it is widely thought that memory problems occur first.
- We want to better understand how dementia affects people with intellectual disabilities and Down syndrome. We explored recent research to see what the early signs of dementia are for people with intellectual disabilities and Down syndrome.
- We found that changes in memory happen early in dementia. We also found that changes in planning skills, attention, mobility, and behaviour might also be early signs of decline.
- Understanding what the early symptoms are, and how they change over time can help improve how health services assess dementia in these populations.

## 1 Introduction

Globally, an estimated 55 million people are living with dementia (World Health Organization 2023). A wealth of research suggests individuals with intellectual disabilities, particularly those with Down syndrome, are at higher risk of dementia than the general population (Coppus et al. 2006; Startin et al. 2016; Strydom et al. 2013). Down syndrome is often considered a genetic form of dementia (Fortea et al. 2021) due to the triplication of chromosome 21, resulting in an overexpression of amyloid‐β in these individuals, increasing their dementia risk (Lott and Head 2019). The reasons for increased dementia risk in individuals with intellectual disability without Down syndrome are less clear but current hypotheses include pre‐existing cognitive abilities, poorer physical health, and higher rates of co‐morbidities e.g., cardiovascular disease (Takenoshita et al. 2023). Premature ageing is also a factor, with ageing in this population beginning as early as age 45 (García‐Domínguez et al. 2020). Although early cognitive changes are well documented (Strydom et al. 2010, 2013; Takenoshita et al. 2020), the exact course of progression, particularly in the early stages, remains poorly understood due to heterogeneity within this population and already compromised baseline abilities (Elliott‐King et al. 2016), as well as approaches to research. Often research has investigated dementia in adults with intellectual disabilities and Down syndrome separately, and far less research is conducted in individuals without Down syndrome (Takenoshita et al. 2020).

In the last two decades, research has shifted focus towards investigating the earliest stages of dementia in people with intellectual disabilities, namely the preclinical and prodromal stages (Krinsky‐McHale and Silverman 2013). The preclinical stage involves biological changes without observable symptoms (Sperling et al. 2013), whilst the prodromal stage is where subtle cognitive and behavioural changes become evident (Scharre 2019). An understanding of these early stages is important to aid early diagnosis. This is crucial when considering that time between diagnosis and death may be as little as three years (Strydom et al. 2010), although this is likely exacerbated by other genetic or health‐related factors such as co‐occurring epilepsy (Hithersay et al. 2019), age at diagnosis, living status (e.g., living with family or in residential settings) (Sinai et al. 2018), and health inequalities (McMahon and Hatton 2021) which limit access to services. For instance, individuals living with family may receive earlier diagnosis due to more consistent care, whereas frequent staff turnover in residential settings may delay recognition (Sinai et al. 2018).

Dementia research for adults with intellectual disabilities and Down syndrome suggests differences in the early stages, in language, functional skills, personality, and behaviour (Deb et al. 2007; Strydom et al. 2010). Cosgrave et al. (2000) investigated dementia symptomology in Down syndrome longitudinally, enabling retrospective examination of symptom onset. They identified memory loss, spatial disorientation, and reduction in activities of daily living (ADLs) as early features, though their sample was limited to individuals with moderate to severe intellectual disabilities. Devenny et al. (2000) attempted to sequence cognitive decline in adults with Down syndrome, comparing those classified as cognitively ‘healthy’ to those with ‘questionable’, ‘early‐stage’, and ‘middle stage’ dementia. They found that early‐stage dementia was associated with poorer visuospatial skills and working memory, whilst short‐term and semantic memory remained intact. However, small sample sizes limit generalisability.

Ball et al. (2006) concluded that frontal lobe functions are implicated earliest, resulting in personality and behavioural changes which may be associated with executive dysfunction. In the general population, gait speed has been linked to global cognitive decline, including executive function (EF) and memory (Hughes et al. 2020; Mielke et al. 2013). It is possible that a similar pattern is also present within intellectual disabilities and Down syndrome. Anderson‐Mooney et al. (2016) proposed that, although changes in gait occur throughout ageing in Down syndrome, a decline in higher‐gait functioning may be more fully explained by dementia‐related pathology, particularly reduced ability in adapting to environmental demands. In addition, they proposed that frontal pathology might also indicate reduced executive abilities; however, the evidence base regarding mobility changes in this population remains sparse.

Two previous reviews have investigated early markers of dementia in this population. Lautarescu et al. (2017) focused on early dementia presentation in Down syndrome, highlighting EF and Behavioural and Psychological Symptoms of Dementia (BPSD) as early indicators, often preceding memory decline. Devshi et al. (2015) focused on non‐cognitive domains, comparing the prevalence of behavioural and psychological symptoms in individuals with intellectual disability not due to Down syndrome to the general population. However, the papers within their review did not explore BPSD prevalence as a whole; instead, each study focused on either behavioural (e.g., pacing, aggression, repetitive movements) or psychological symptoms (e.g., apathy, anxiety, depression) in isolation. Both reviews noted methodological shortcomings of included studies, such as the availability and use of appropriate measures for this population, and suggested factors such as dementia classification criteria and validity of measures as accounting for discrepancies between studies. Nevertheless, there remains a lack of reviews encompassing both cognitive and non‐cognitive aspects of functioning, such as mobility and functional skills. Since these reviews were published, more research into the early markers of dementia in these populations has emerged, highlighting the need for an updated, comprehensive synthesis.

## 2 Aim of the Review

This review sought to explore the early symptoms of dementia in people with intellectual disabilities and to consider the trajectory of decline in functioning. A holistic view is required to help identify clinical changes rather than viewing symptoms in discrete silos, as is often the case. Failure to fully understand the symptomology and early presentation of dementia has implications for diagnosis and management (Cipriani et al. 2018). Despite dementia being a national priority, individuals with intellectual disabilities are often neglected in policy (Burke et al. 2018). It is key for there to be a clearer understanding of the early presentation of dementia in this population to inform clinical practise. Using narrative synthesis to explore recent research into early symptoms of dementia for this population, the present review encompasses cognitive aspects, including memory, language, and EF, in addition to non‐cognitive domains such as behaviour, mood, and mobility. This review contrasts previous reviews by combining literature exploring a wide range of cognitive and non‐cognitive domains. The results will be presented and critically evaluated, with implications for clinical practise and future research discussed.

## 3 Materials and Methods

A systematic review was conducted using the Preferred Reporting Items for Systematic Reviews and Meta‐Analyses Statement (PRISMA; Page et al. 2021). The review was registered on PROSPERO in December 2023 (CRD42023480122).

### 3.1 Search Strategy

**FIGURE 1:**
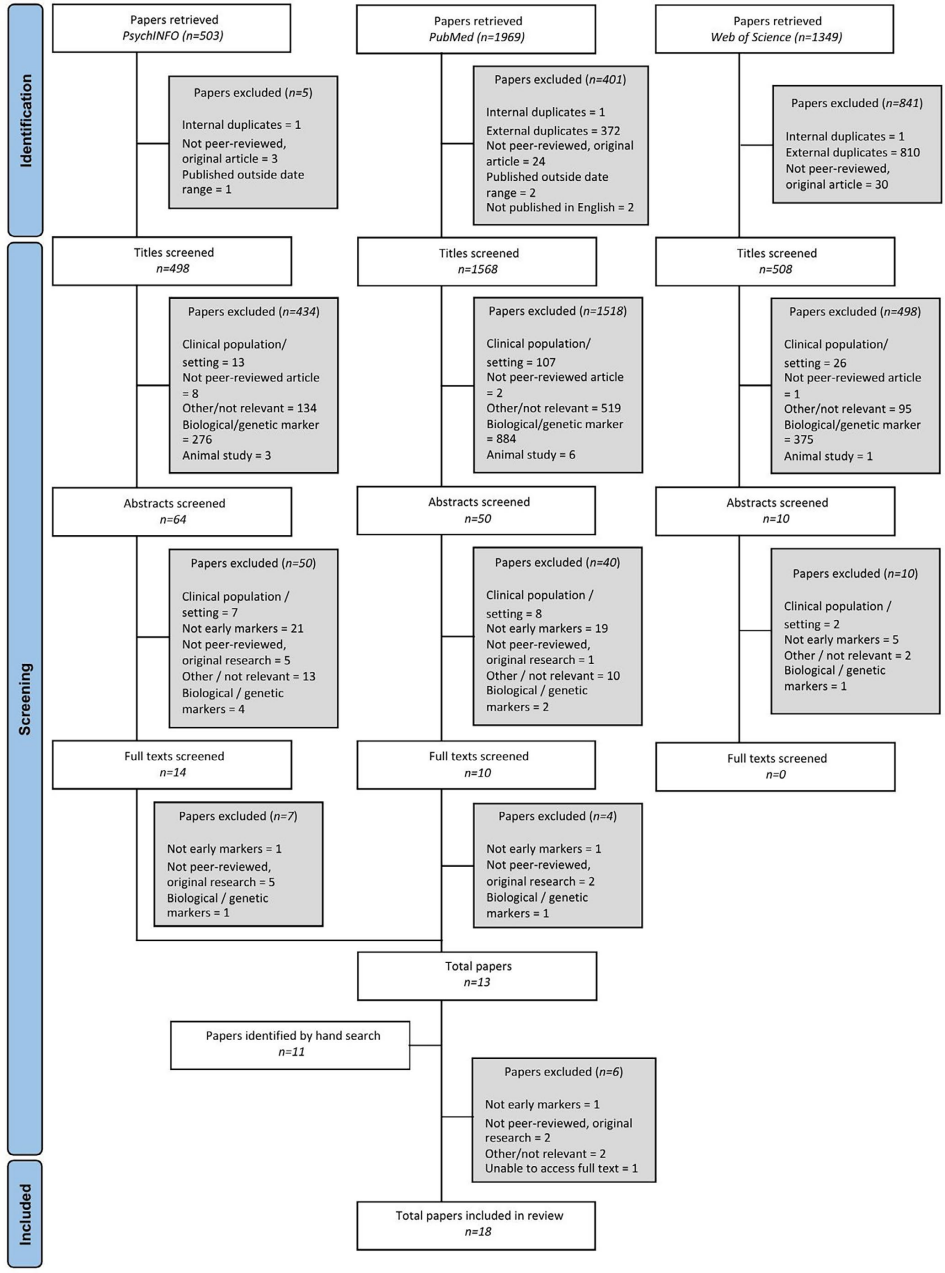
Flowchart of screening process for selected studies.

Online databases (PsychInfo, PubMed, Web of Science) were searched for articles published between 1st January 2015 and 17th November 2023. Articles from 2015 onwards were included to avoid duplication of previous reviews in this area. Searches included the following keywords: “intellectual disabilities,” “dementia,” and “symptoms.” Boolean operators and truncations were applied. The full search strategy is available as Supporting Information. Search terms were kept broad to encompass a wide range of literature, as well as to be in keeping with similar reviews. Additionally, a hand search of reference lists of included studies was undertaken. A systematic selection process was employed, which involved screening titles, abstracts, and full text articles (Figure 1).

### 3.2 Inclusion and Exclusion Criteria

The inclusion criteria were as follows: papers were empirical, published in peer‐reviewed journals, written in English, and recruited a sample of individuals with intellectual disabilities and/or Down syndrome. The studies' focus was on identifying early signs of dementia in this population; however, participants were not required to have a formal dementia diagnosis. The exclusion criteria were animal studies, studies exploring biological or genetic markers, and studies describing dementia symptoms generally (i.e., not early stages).

Searches and initial screening were performed by author JD. Any duplicates detected by referencing software were screened and manually removed by JD. The following two screening stages (titles/abstracts and full texts) employed a second reviewer (AM) to independently review a proportion of the articles (20%). Disagreements were resolved by a third reviewer, author WD. Screening decisions were recorded and compared using the Rayyan software for systematic reviews (Ouzzani et al. 2016).

### 3.3 Quality Assessment

Articles were quality assessed using the Standard Quality Assessment Criteria (QualSyst) tool (Kmet et al. 2004). The QualSyst was chosen over other appraisal tools due to its applicability to cross‐sectional and longitudinal studies, and inclusion of both quantitative and qualitative checklists, enabling use across methodologies. Quality assessments were completed by author JD, with 20% rated by a second reviewer (AM). The QualSyst provides a checklist of criteria which enable the calculation of a quality summary score. Scores closer to one indicate higher quality studies. Summary scores were interpreted using the following criteria: strong (≥ 0.80), good (0.70–0.79), adequate (0.50–0.69), and poor (< 0.50). There are no set guidelines on inclusion thresholds; however, Kmet et al. (2004) provide suggestions for liberal and conservative cut‐offs based on summary scores. A liberal cut‐off of 0.55 was applied.

### 3.4 Data Extraction

Data extraction was completed by author, JD. For each study, information regarding the following was extracted: study location, population, sample size and method, sample characteristics (age, gender, level of intellectual disability), grouping of participants based on dementia status, methods of classifying dementia diagnosis, domains assessed, measures used, and relevant findings relating to early symptoms.

### 3.5 Data Analysis

Narrative synthesis was conducted to explore and compare study findings. This approach was chosen instead of meta‐analysis due to the heterogeneity of included studies, specifically the measures used and functions examined. The synthesis was based on methods proposed by Popay et al. (2006), utilising four key elements: development of theory, preliminary synthesis of findings, exploration of relationships within the data, and assessing the robustness of the synthesis.

## 4 Results

Initial searches yielded a total of 3821 papers which were screened for duplicates and papers which violated the limiters, reducing this to 2574 papers. Additional papers (*n* = 11) were identified through hand searches and screened using the same criteria. The final review consisted of 18 papers. Descriptive characteristics are summarised in Table 1, with an overview of the key findings presented in Table 2.

**TABLE 1:** Descriptive characteristics of included studies.

Citation		Country	Sample size (*N*)	Participant characteristics (diagnosis, age, gender, sampling method)	Dementia groups included	Method for classifying dementia diagnosis	Study design
1	Arvio and Bjelogrlic‐Laakso (2021)	Finland	230	Individuals with intellectual disability, age ≥ 34 years. 32 Down syndrome, 19 cerebral palsy‐intellectual disability syndrome, 19 other genetic syndromes.	8 individuals with Down syndrome diagnosed with dementia prior to study	Diagnosed prior to study	Cross‐sectional design with no comparator group
				Mean age: 51 years			Quantitative methodology
				Gender: 102 (44.3%) females			
				Sampling method: purposive			
2	Aschenbrenner et al. (2021)^a^	UK, Spain, US	312	Individuals with Down syndrome, age ≥ 35 years with at least 2 previous assessment points. No diagnosis at baseline visit.	N/A	Not specified in study methodology	Multi‐centre, retrospective longitudinal design with no comparator group
				Mean age: 44.5 years			Quantitative methodology
				Gender: 150 (48.1%) females			
				Level of intellectual disability			
				Mild: 113 (36.2%)			
				Moderate: 144 (46.2%)			
				Severe: 46 (14.7%)			
				Sampling method: purposive. Secondary analysis of pre‐existing data from multiple cohorts.			
3	Benejam et al. (2015)	Spain	90	Individuals with Down syndrome, age ≥ 18 years. Absence of significant language or sensory impairment, mental health disorders, and cognitive or functional decline.	Stable cognition (*n* = 75)	Retrospective screening	Single‐centre, cross‐sectional design. Inclusion of dementia comparator group
				Mean age: 36.1 years			
				Gender: 42 (46.7%) females	DS‐DAT (*n* = 15)		Quantitative methodology
				Sampling method: purposive			
4	Benejam et al. (2020)^b^	Spain	343	Individuals with Down syndrome, age ≥ 18 years. Excluded if any psychiatric or medical disorder which could affect cognition.	Asymptomatic (*n* = 277), prodromal dementia (*n* = 31), dementia (*n* = 35), uncertain (excluded from analysis)	Classified by both neurologists and neuropsychologists	Single‐centre, cross‐sectional design. Dementia comparator group
				Median (IQR) age: 41 (18.5)			
				Gender: 169 (49.0%) females			
				Level of intellectual disability			
				Mild: 91 (26.5%)			Quantitative methodology
				Moderate: 205 (59.8%)			
				Severe + profound: 47 (13.7%)			
				Sampling method: purposive			
5	Blok et al. (2017)	Netherlands	68	Individuals with Down syndrome, excluding those who were non‐verbal, or had visual or hearing loss. Participants lived in a regional institution for individuals with intellectual disability. The average level of intellectual ability was judged to be moderate (exact IQ scores not known).	None	Not specified in study methodology	Cross‐sectional design with no comparator group
				Mean (SD) age: 43.4 (9.5)			
				Gender: 28 (41.2%) females			Quantitative methodology
				Sampling method: purposive, analysis of secondary data			
6	Conceição et al. (2023)	Brazil	66	Individuals with Down syndrome, age ≥ 20 years. Excluded if presence of mental health or medical conditions which may influence cognition, or severe gait impairment requiring assistive devices to walk.	Stable (*n* = 36), Prodromal (*n* = 10), Dementia (*n* = 20)	Classified by clinicians utilising neuropsychological tests (CAMCOG‐DS, CAMDEX‐DS) using multidisciplinary approach.	Single‐centre, cross‐sectional design utilising between group comparisons
				Mean (SD) age: 40.9 (10.6)			Quantitative methodology
				Gender: 28 (42.2%) females			
				Level of intellectual disability			
				Mild: 30 (45.4%)			
				Moderate: 24 (36.4%)			
				Severe: 7 (10.6%)			
				Unspecified: 5 (7.6%)			
				Sampling method: purposive			
7	Dekker et al. (2021)^c^	Netherlands, Belgium, France, Italy, Spain	524	Individuals with Down syndrome, age ≥ 30 years recruited through care institutions. Excluded individuals with profound intellectual disability, long‐term admission to hospital in past 6 months, end of life care, recent significant life event or cardiovascular event, or behavioural changes related to another condition.	No dementia (*n* = 292), Questionable dementia (*n* = 119), Clinically diagnosed dementia (*n* = 113)	Diagnosis established prior to study based on clinical judgement using multidisciplinary evaluation, informant interview(s) and medical reports	Cross‐sectional design utilising between group comparisons Quantitative methodology
				Mean age: 52.8			
				Gender: 246 (46.9%) females			
				Level of intellectual disability			
				Mild: 61 (11.6%)			
				Moderate: 334 (63.7%)			
				Severe: 129 (24.6%)			
				Sampling method: purposive			
8	Firth et al. (2018))	UK	283	Individuals with a clinical diagnosis of Down syndrome, age ≥ 16 years, living in family homes and residential facilities. Excluded if presence of acute physical or mental health condition. Participants split into two groups based on age—young adults, those aged 16–35 (*n* = 119) and older adults, those aged 36 and older (*n* = 164) groups.	Dementia (*n* = 43), no dementia (*n* = 240)	Based on pre‐existing clinical diagnosis after comprehensive clinical assessment	Cross‐sectional design. Young adult (aged 16–35 years) group used as control group
				Mean age: 37.4			
				Gender: 139 (49.1%) females			
				Level of intellectual disability			
				Mild: 114 (40.3%)			Quantitative methodology
				Moderate: 130 (45.9%)			
				Severe: 39 (13.8%)			
				Sampling metho: purposive			
9	Fonseca et al. (2020)	UK, Brazil	162	Individuals with Down syndrome, age ≥ 30 years.	Stable cognition group (*n* = 114), prodromal dementia (*n* = 24), dementia (*n* = 24)	Established using CAMDEX‐DS, ICD‐10 and DSM‐IV criteria, alongside informant interviews	Cross‐sectional design utilising between group comparisons
				Mean (SD) age: 42.5 (8.2)			
				Gender: 69 (42.6%) females			Quantitative methodology
				Sampling method: purposive. Secondary analysis of pre‐existing data from multiple cohorts.			
10	García‐Alba et al. (2019)	Spain	55	Individuals with Down syndrome, age ≥ 40 years with genetically confirmed karyotype excluding mosaicism and translocations (*n* = 41). All presented with mild or moderate intellectual disability (breakdown not reported).	Stable cognition (*n* = 14), MCI (*n* = 14), dementia (*n* = 13)	Categorises determined based on participants meeting criteria for MCI or AD, or not. Criteria described in paper	Cross‐sectional design utilising between group comparisons
				Mean age: 49.9			Quantitative methodology
				Gender: 25 (61.0%) females			
				Control group: healthy adults without Down syndrome (*n* = 14) matched in age and gender with the CN‐DS group.			
				Mean age: 45.2			
				Gender: 10 (71.4%) females			
				Sampling method: purposive			
11	Hartley et al. (2020)^d^	UK, US	118	Individuals with Down syndrome, age ≥ 25 years. Participants were requited to have 2 or more data collection time points between 2010 and 2019.	Stable cognition, MCI, dementia, unable to determine	Diagnostic consensus including at least 3 clinicians, considering medical history, informant reports and ratings, evaluation of adaptive skills and direct assessment	Longitudinal design. Data collected over 5 time points. No comparator group
				No clinical diagnosis at time point 1.			Quantitative design
				Mean (SD) age: 37.2 (7.7)			
				Gender: 61 (52%) females			
				Sampling method: purposive. Participants were part of an already established longitudinal multi‐site study			
12	Hom et al. (2021)	US	144	Individuals with Down syndrome, age ≥ 40 years (range: 40–81). Chromosomal diagnosis included: trisomy 24 (84.0%), mosaic (4.9%), translocation (3.5%), and unknown (7.7%).	Stable cognition group (*n* = 103) & MCI (*n* = 41)^e^	Comprehensive assessment and consensus review by staff at study sites	Cross‐sectional design utilising between group comparisons
				Participants were included if they had estimated pre‐existing IQ > 30 and did not have any significant sensory or motor impairments.			
				Mean age: 50.8			
				Gender: (41%) females			
				Level of intellectual disability			Quantitative methodology
				Mild: 53.5%			
				Moderate: 46.5%			
				Sampling method: purposive			
13	Mgaieth et al. (2023)	UK	302	Individuals with Down syndrome, age ≥ 16 years with mild to moderate intellectual disability.	Dementia and no dementia	Completed by specialists using comprehensive assessments and medical records. Assessments included cognition and adaptive functioning	Longitudinal cohort study utilising between group comparisons
				302 at baseline and 87 at follow‐up.			
				Participants split into two groups based on age—young adults, those aged 16–35 (*n* = 132) and older adults, those aged 36 and older (*n* = 170) groups.			
				Mean age of total sample: 37.8			
				Mean age (SD) per age group: YA = 26.1 (5.4), OA = 49.6 (8.0)			
				Gender: 152 (50.3%) females			Quantitative methodology
				Level of intellectual disability			
				Mild: 134 (44.4%)			
				Moderate: 168 (55.6%)			
				Sampling method: purposive			
14	Pulsifer et al. (2020)	US	168	Individuals with Down syndrome with karyotype of full trisomy 21, age ≥ 40 years with estimated pre‐existing IQ ≥ 30.	Stable cognition (57.8%), MCI (22.6%), probable/definite dementia (19.6%)	Clinical status determined via consensus review by staff and researchers who had direct contact with individual, use of assessment results, and pre‐existing level of cognitive functioning	Cross‐sectional design utilising between group comparisons
				Mean (SD) age: 51.4 (7.1)			
				Gender: 72 (42.9%) females			
				Level of intellectual disability			
				Mild: 85 (50.6%)			Quantitative methodology
				Moderate: 73 (43.4%)			
				Severe: 10 (6.0%)			
				Sampling method: purposive			
15	Startin et al. (2019)	UK	297	Individuals with Down syndrome, age ≥ 16 years.	Participants aged ≥ 36 split into	For those 36+ years and no clinical diagnosis prior to the study, 2 psychiatrists reviewed CAMDEX‐DS scores	Cross‐sectional design utilising between groups comparisons
				Mean age: 42.2			
				Gender: 148 (49.8%) females			
				Level of intellectual disability			
				Mild: 114 (38.4%)	Preclinical (*n* = 66), prodromal (*n* = 44), clinical (*n* = 45), missing (*n* = 18)		Quantitative methodology
				Moderate: 135 (45.5%)			
				Severe: 48 (16.2%)			
				Sampling method: purposive			
16	Van Pelt et al. (2020)	US	28	Individuals with Down syndrome age ≥ 25 years residing in the community. Karyotype of trisomy 21 with stable general medical condition and medications for at least 3 months prior to study.	No dementia (*n* = 22), other/possible dementia (*n* = 6)	NINCDS‐ADRDA AD criteria used by panel of neurologists and neuropsychologists to reach consensus diagnosis	Cross‐sectional design utilising between group comparisons
				Mean (SD) age: 36.6 (7.0)			Quantitative methodology
				Gender: 13 (46.4%) females			
				Level of intellectual disability			
				Borderline/mild: 16 (57.1%)			
				Moderate: 12 (42.9%)			
				Sampling method: purposive			
17	Wissing et al. (2022)^f^	Netherlands	Survey: 100	Care professionals and family members of people with intellectual disabilities and suspected dementia.	Suspected dementia	Not reported	No comparator group
			Interviews: 16	Care professionals, *n* = 87			Mixed methods
				Family members, *n* = 13			
				Care professionals with vast experience in signalling/diagnosing dementia in people with severe/profound intellectual disability.			
				Sampling method: purposive			
18	Wissing et al. (2023)	Netherlands	141	Individuals with severe, severe to profound, or profound intellectual disabilities, with or without Down syndrome, age ≥ 40 years. All participants lived in residential facilities.	No dementia (*n* = 103), questionable dementia (*n* = 19), dementia (*n* = 19)	Data extracted from clinical records	Cross‐sectional retrospective analysis of clinical records utilising between group comparisons
				Mean age: 63.7			Quantitative methodology
				Gender: not reported			
				Level of intellectual disability			
				Severe: 81 (57.4%)			
				Severe/profound: 4 (2.8%)			
				Profound: 56 (39.7%)			
				Sampling method: purposive			

Abbreviations: AD, Alzheimer's disease; CAMCOG‐DS, Cambridge Cognitive Examination for Older Adults with Down syndrome; CAMDEX‐DS, Cambridge Examination for Mental Disorders of Older People with Down syndrome and Others with Intellectual Disabilities; DS‐DAT, Down syndrome‐Dementia of the Alzheimer's Type; DSM‐IV, diagnostic and statistical manual of mental disorders‐Fifth Edition; IQR, inter‐quartile range; ICD‐10, International Classification of Diseases, Tenth Revision; MCI, mild cognitive impairment; N/A, not applicable; NINCDS‐ADRDA, National Institute of Neurological and Communicative Diseases and Stroke/Alzheimer's Disease and Related Disorders Association; OA, older adults; SD, standard deviation; YA, young adults.

^a^ This paper contains details of five study sites across three countries, which each administered different test batteries across different cognitive domains. The combined study characteristics are summarised here.

^b^ This paper presented descriptive statistics as medians and interquartile ranges.

^c^ This study was conducted with informants rather than directly with people with Down syndrome. The 954 informants completed the measures for 524 individuals with Down syndrome. As such, 186 interviews were conducted with one informant, 250 were conducted with two informants, and 88 were conducted with three informants. Demographics are reported for the individuals with Down syndrome.

^d^ This study was completed over 5 time points between 2009 and 2019. Data for time point 1 is presented.

^e^ Only data for the stable cognition and MCI groups were analysed in this study.

^f^ This study reported descriptive statistics for informants completing the surveys and, as such, are not reported here.

**TABLE 2:** Summary of key findings of included studies.

Citation		Cognitive domains assessed	Non‐cognitive domains assessed	Key findings
1	Arvio and Bjelogrlic‐Laakso (2021)	Orientation, forgetfulness, speech	Mood, personality, functional skills, social interaction, behaviour	Common dementia symptoms
				Loss of energy, self‐care skills, forgetfulness, and variable mood.
				No age‐related differences
2	Aschenbrenner et al. (2021)	Language, visuospatial ability, memory, orientation, executive function, attention, praxis, perception	N/A	Cognitive domains sensitive to early decline
				Memory, Language, Selective attention, and Praxis.
3	Benejam et al. (2015)	Memory (free and cued recall)	N/A	Healthy Down syndrome group
				↓ mCRT scores with age‐poorer recall and greater number of errors.
				Down syndrome with dementia group
				Poorer performance across all mCRT domains.
				Down syndrome with early‐stage dementia showed little benefit from cueing, indicating difficulties with storage of information.
4	Benejam et al. (2020)	Memory, attention, language, orientation, praxis, abstract thinking, perception	N/A	↓ in CAMCOG‐DS and mCRT scores along the dementia continuum.
				Sig. differences between mild and moderate groups in asymptomatic Down syndrome but not prodromal Down syndrome.
5	Blok et al. (2017)	Episodic memory	Temperament, adaptive functioning	↓ across all domains. Earliest deterioration found for episodic memory.
				No evidence of changes in adaptative functioning or personality occur before changes in memory.
6	Conceição et al. (2023)	Orientation, language, memory, attention, praxis, abstract thinking, perception	Balance, gait	↓ gait performance as a predictor of prodromal dementia and clinical dementia.
7	Dekker et al. (2021)	N/A	BPSD	Changes in frequency and severity of
				Anxious, sleep‐related, restless, and stereotypic, irritable, apathetic, depressive, and eating/drinking behaviour
				Higher frequency and severity of behaviours in the dementia group, lowest in Down syndrome group.
				1/3+ of questionable dementia group showed ↑ in anxious, sleep‐related, irritable, apathetic, and depressive behaviour
				These behaviours may be early signs of AD in Down syndrome
8	Firth et al. (2018)	Visuospatial memory, object memory, orientation, rule learning and set shifting, working memory, planning, semantic verbal fluency, attention	Motor ability, co‐ordination, adaptive skills, social skills	Early ↓ in memory, sustained attention, motor co‐ordination, and verbal fluency.
				Changes in executive abilities occur later with behavioural changes observed by informants occurring last.
				Test of memory and sustained attention most useful for tracking decline in preclinical/prodromal stages.
9	Fonseca et al. (2020)	Executive dysfunction	Disinhibition, apathy	Prodromal group showed ↓ memory and executive skills compared to stable cognition group.
				Deterioration begins with memory deficits and executive dysfunction (prodromal)
				Disinhibition and apathy manifest later
				More amnestic initial presentation in the presence of earlier frontal changes in Down syndrome compared to general population.
10	García‐Alba et al. (2019)	Memory, executive function, orientation	N/A	Transition from MCI to AD characterised by ↓ global cognition, ↑ temporal disorientation, and marked amnesic deficit.
				MCI group ↓ performance on CAMCOG‐DS, DVM, TO, WM, and ADVM measures compared to stable cognition group—possible cognitive markers of MCI in Down syndrome.
				Dementia group performed ↓ on verbal and working memory compared to MCI group.
11	Hartley et al. (2020)	Episodic memory, visual attention, executive functioning	Motor planning, co‐ordination	The Cued Recall Test emerged as a promising indicator of transition from preclinical to prodromal AD.
12	Hom et al. (2021)	Language, executive function, memory, visuomotor skills.	N/A	Language/executive abilities, memory and visuomotor skills predicted MCI status. Differences based on modelling system used
				Path modelling—language/executive most affected by prodromal AD
				Structural equation modelling—memory scores impacted first, followed by visuomotor scores, followed by language/executive scores.
13	Mgaieth et al. (2023)	Verbal fluency	N/A	Semantic verbal fluency observed as an early indicator of cognitive deterioration with ↓ words produced and ↑ intrusion errors.
14	Pulsifer et al. (2020)	Language, verbal fluency	N/A	Assessment of language skills aided early detection of cognitive decline
				Receptive language a predictor of MCI.
				Semantic verbal fluency was identified as the strongest predictor of AD.
				Informant reports are able to identify early stages (from stable cognition to MCI).
15	Startin et al. (2019)	Memory, executive function	Motor co‐ordination	Changes in memory and attention identified as domains most sensitive to progression from preclinical to prodromal dementia.
				Prodromal group performed ↓ on several memory, executive and attentional measures than the preclinical group.
				Memory and attention measures most sensitive to progression from pre‐clinical to prodromal stage.
16	Van Pelt et al. (2020)	N/A	Gait	↑ dual‐task effects on gait velocity based on dementia diagnosis. No association between dementia and velocity, step length or base width.
				Dual‐task gait may serve as an indicator of early‐stage dementia in Down syndrome.
17	Wissing et al. (2022)	Memory, planning, problem solving, orientation, visuospatial skills	BPSD, motor function	Most frequently observed symptoms included ↓ in
				ADL functions and BPSD (irritability, eating/drinking, anxious and apathetic behaviour).
				Cognitive and motor changes were observed to a lesser extent.
18	Wissing et al. (2023)	Memory, orientation, language, object recognition, executive skills, visuospatial	ADLs, BPSD, motor function	↑ frequency of symptoms for those with diagnosed or questionable dementia with most prevalent early symptoms being
				↓ memory and mobility
				↑ anxious, apathetic, and irritable behaviour.

Abbreviations: AD, Alzheimer's disease; ADL, activity of daily living; ADVM, auditory delayed verbal memory; BPSD, behavioural and psychological symptoms of dementia; CAMCOG‐DS, Cambridge cognitive examination for older adults with down syndrome; DVM, delayed visual memory; MCI, mild cognitive impairment; mCRT, modified Cued recall test; N/A, not applicable; TO, temporal orientation test; WM, working memory.

### 4.1 Quality of Included Studies

Quality assessment ratings are available as Supporting Information. Inter‐rater agreement ranged from 71% to 86%. Most discrepancies reflected differences of opinion on the assignment of ‘Yes’ versus ‘Partial’ responses. All studies received ratings above the 0.55 cut‐off. For quantitative papers, two were rated as adequate, seven were rated as good, and eight were rated as strong. The mixed methods paper was rated as good and above. All studies stated the objective; this was not always explicit but could be inferred and drew conclusions consistent with the reported results. Reasons for lower scores were related to study design, lack of comparator groups, and limited control for confounding variables, with only a selection of studies controlling for age and level of intellectual disability. Few studies provided clear estimates of variance, and the mixed methods paper failed to discuss research reflexivity.

### 4.2 Descriptive Characteristics

Numerous studies exploring the early symptoms of dementia in the intellectual disability and Down syndrome populations have been conducted since 2015, focusing on cognitive and non‐cognitive factors. Four studies were multi‐national studies (2, 7, 9, 11) including the United Kingdom, Spain, United States, Netherlands, Belgium, France, Italy, and Spain. Of the single nation studies, most were conducted in the United Kingdom (8, 13, 15, *n* = 3), United States (12, 14, 16, *n* = 3), the Netherlands (5, 17, 18, *n* = 3), and Spain (3, 4, 10, *n* = 3). Individual studies were conducted in Brazil (6) and Finland (1). Overall, most studies were conducted in Western countries.

The included studies recruited a total of 3176 participants with either intellectual disability or Down syndrome, whilst one study recruited 100 care professionals and family members of individuals with intellectual disability instead (17). The majority of studies recruited adults with Down syndrome (*n* = 15). The remaining three studies recruited samples of individuals with intellectual disability with or without Down syndrome. Inclusion criteria regarding age varied widely across studies, from any age/≥ 16 years (*n* = 5), ≥ 18 years (*n* = 2), ≥ 20 years (*n* = 1), ≥ 25 years (*n* = 2), ≥ 30 years (*n* = 2), ≥ 34 years (*n* = 1), ≥ 35 years (*n* = 1), and ≥ 40 years (*n* = 4). The reported mean age of participants ranged from 36.6 to 69.9 years with an overall mean of 46.6 years (SD = 9.6). Two studies did not report the mean age of participants and therefore were excluded from the summary statistics. Age ranges were not consistently reported. Eleven studies reported the levels of intellectual disability, which encompassed borderline, mild, moderate, severe, and profound.

Sample sizes ranged from 28 to 524 participants (*M* = 189.8, SD = 131.0, median = 153). As might be expected, multi‐site studies typically had larger samples; however, many single‐site studies achieved samples greater than 200 (*n* = 5). The majority of studies (*n* = 16) reported information regarding gender. Of these, the female to male ratio was approximately equal (female *M* = 48.22%, SD = 5.35, range 41.2%–61%). Included studies used a range of methodologies with a bias towards exploring cognitive functions. Nearly all studies utilised a non‐experimental cross‐sectional design (*n* = 14), whilst a small proportion adopted a longitudinal design (*n* = 3) or mixed methods (*n* = 1). Of the included studies, most conducted between‐group comparisons (*n* = 14) with a combination of comparisons to control groups, between dementia categories, or both. Both cognitive and non‐cognitive domains were explored in nine studies; only cognitive domains were explored in seven studies, and only non‐cognitive domains were explored in two studies.

### 4.3 Dementia Diagnosis

Many studies categorised participants based on already established dementia diagnosis, or retrospective diagnosis using clinical judgement and/or neuropsychological evaluation (1, 3, 4, 6, 7, 8, 9, 10, 11, 12, 13, 14, 15, 16, 18). Although this enables comparisons between groups (stable cognition, preclinical, prodromal, and clinical), which can be helpful in considering differences in functioning, the definitions of these categories varied between studies or were ambiguous. Ten studies included groups that were categorised under three main domains (4, 6, 7, 9, 10, 11, 12, 14, 15, 18): stable cognition, suspected dementia, and clinical dementia. The stable cognition group included preclinical participants without dementia and was broadly defined as the absence of decline, no decline beyond normal ageing, or asymptomatic. The suspected dementia group included prodromal and probable dementia and is defined by the presence of symptoms that do not meet diagnostic criteria. Finally, the clinical dementia group included those where diagnostic criteria are met. Three studies also included an ‘uncertain’ or ‘missing’ group where dementia status could not be determined (11, 12, 15); however, these individuals were excluded from the analysis. One study included only suspected dementia (17). These variations complicate comparisons across studies.

Approaches to determining dementia status are included in Table 2, with varying degrees of detail provided. Several studies used clinician judgement based on opinions from neurologists and neuropsychologists reviewing medical records, whereas other studies included the use of standardized assessments such as the CAMDEX‐DS and informant‐based measures such as the Dementia Questionnaire for People with Learning Disabilities. The lack of inclusion of standardized tests increases the subjectivity associated with dementia groups and increases the risk of bias. Several studies failed to include any comparator group, which limits the inferences that can be drawn regarding early symptoms.

### 4.4 Cognitive and Non‐Cognitive Measures

**FIGURE 2:**
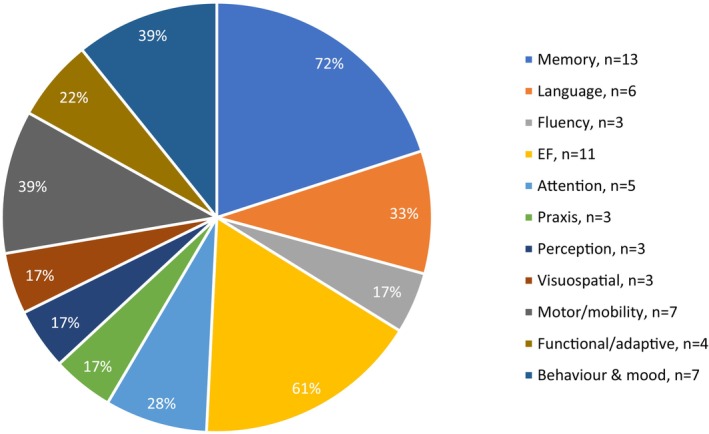
Frequency of domains assessed across included studies and percentages of studies assessing each domain.

A range of measures was used to assess cognitive and non‐cognitive functions. An overview of these is available as Supporting Information. Overall, 66 different measures were used, including direct assessment batteries (*n* = 7), tests (*n* = 11), tasks (*n* = 37), scales (*n* = 1), and informant‐based measures (*n* = 10). Figure 2 shows the proportions of domains assessed across the studies. Generally, there was a bias towards assessing memory, with 13 studies (72%) exploring aspects of memory, closely followed by EF with 11 studies (61%) examining this. Far fewer studies explored BPSD, perception, visuospatial abilities, praxis, and fluency.

### 4.5 Cognitive Domains

A range of cognitive domains was assessed including language, memory, orientation, EF, visuospatial skills, praxis, perception, rule learning, and verbal fluency.

#### 4.5.1 Memory

Eleven studies suggested that memory changes indicate early decline. However, whilst some examined memory as a broad construct, others focused on specific processes within memory, such as episodic memory, recognition, cued recall, immediate memory, auditory memory, and visual memory. When considering memory broadly, the findings suggest memory performance declines across the AD continuum and that changes occur early, distinguishing those with prodromal dementia from asymptomatic individuals. Benejam et al. (2015) concluded that memory changes were characterised by poorer recall, increased intrusion errors, and limited benefit from cueing, indicative of a storage deficit. Episodic memory was frequently cited as the earliest marker of decline in Down syndrome (Blok et al. 2017; Hartley et al. 2020), along with immediate memory (Blok et al. 2017), visual and verbal memory (García‐Alba et al. 2019). One study found that adults with Down syndrome with higher amyloid‐β burden demonstrated poorer episodic memory and greater decline over time (Hartley et al. 2020). However, Benejam et al. (2015) noted that memory changes were observed in individuals with Down syndrome regardless of the presence of dementia, concordant with previous research indicating that some degree of memory decline is expected with normal ageing (Nagdee 2011). However, it is important to consider whether these changes reflect mild cognitive impairment with possible transition into dementia as opposed to normal cognition (Shi et al. 2014).

The large variation in memory measures used is problematic. Several studies used the modified Cued Recall Test, which is specifically designed to assess verbal episodic memory in Down syndrome and demonstrates good validity and sensitivity to prodromal dementia (Krinsky‐McHale et al. 2022). Other studies used batteries designed for the intellectual disabilities/Down syndrome populations (e.g., CAMCOG‐DS), or memory subtests from these (e.g., Cued Recall taken from the NEPSY‐II). Measures developed for the general population were used in other studies, e.g., the Rivermead Behavioural Memory Test, which lacks validation for the intellectual disability population, raising concerns about accessibility, available normative data, and construct validity.

#### 4.5.2 Executive Function

EF was the second most frequently assessed domain across the included studies. Three studies reported early EF impairments, with participants in the prodromal groups demonstrating poorer performance on EF measures than those at preclinical stages. When considering specific constructs of EF, García‐Alba et al. (2019) found working memory deficits in the prodromal group compared to controls. However, this deterioration was less marked than delayed visual memory impairments, suggesting deficits in storage and retrieval, rather than manipulation of information. Other studies found deterioration in these skills occurred later, with planning, set‐shifting, and disinhibition all occurring following memory changes (Firth et al. 2018; Fonseca et al. 2020). Hom et al. (2021) reported that several EF measures had to be removed from the analysis due to high error rates, likely caused by difficulties with comprehension of task instructions, and participants therefore being unable to complete the tasks. Some EF measures were also found to demonstrate weak loadings onto the EF domain, or loadings onto multiple factors (e.g., language), leading to their removal from the analysis. Despite this, the findings still support EF decline during the prodromal stage.

Whilst several studies identified EF decline as an early marker, Firth et al. (2018) argued that even though executive dysfunction may be present within the prodromal stage, these are not the first changes observed. Fonseca et al. (2020) similarly concluded that memory deficits better distinguished prodromal dementia from stable cognition than EF. They concluded that there is an initial amnestic presentation; however, this occurs in the presence of earlier frontal changes than in the general population.

#### 4.5.3 Attention

Two of the included studies reported attention to be an early indicator of decline. Aschenbrenner et al. (2021) suggested that selective attention was sensitive to early change; however, they only used a single test (cancellation) to measure attentional abilities. Conversely, Firth et al. (2018) concluded that sustained attention was more useful in tracking decline. Through event sequencing models, they found multiple areas of cognition were implicated as being associated with early decline indicative of dementia pathology. Although they noted that many of these domains were underpinned by attentional abilities, rather than the results directly indicating a role of attention in the early stages of dementia, they deduced that these attentional difficulties may be an underlying factor in decline in other areas.

#### 4.5.4 Language and Verbal Fluency

Verbal fluency is often considered an EF construct; however, a selection of studies specifically evaluated verbal fluency across stages of dementia. Verbal fluency was reported by two studies as an early marker and the strongest predictor of clinical dementia status in another study. Three studies reported language to be an early indicator, with one study specifying that receptive language was the best predictor (Pulsifer et al. 2020). Changes across language and verbal fluency were characterised by poorer recall and increased intrusions. Similarly to memory and EF, modelling approaches differ in their outcomes regarding language, with one approach suggesting language is more impacted than memory in the prodromal stage, whilst other approaches suggest the opposite. In later stages, semantic verbal fluency appears to be a strong predictor of clinical dementia (Pulsifer et al. 2020).

#### 4.5.5 Other Cognitive Domains

Additionally, there were single instances of praxis, temporal orientation, and visuomotor skills being reported as early indicators. Hom et al. (2021) concluded that, although lower scores on measures of visuomotor skills are associated with prodromal dementia, these skills are affected to a lesser extent than memory, language, and EF. Similarly, García‐Alba et al. (2019) concluded that although poorer temporal orientation was observed in those with prodromal dementia and likely characterised the transition into clinical dementia, verbal and working memory decline was more significant in the early stages.

### 4.6 Non‐Cognitive Domains

The non‐cognitive domains explored within the included studies can be divided into three groups: mobility/motor functions, which included gait and motor co‐ordination; behaviour/personality characteristics, which included BPSD; and functional skills, including ADLs.

#### 4.6.1 Mobility and Motor Skills

Three studies suggested that motor functions were impaired in the early stage of dementia, particularly reduced gait, mobility, and motor coordination. Although most studies exploring mobility/motor symptoms also assessed cognitive abilities, these were often reported separately and therefore it is unclear whether mobility changes occurred alongside, before, or after changes in cognition. Conceição et al. (2023) suggested that memory and scores on mobility measures were strongly correlated, such that memory deterioration was associated with declines in gait performance (initiation, symmetry, and continuity), with gait being a significant predictor of prodromal dementia. Wissing et al. (2023) concluded that both prodromal and clinical dementia groups demonstrated more motor symptoms than those without dementia, with the prodromal group being characterised by a high prevalence of reduced walking skills and stiffness, as well as decreased balance being common. Decline in motor coordination was also considered in the early stages of dementia (Firth et al. 2018). Other papers suggested that motor changes are observed to a lesser extent than other non‐cognitive symptoms (Wissing et al. 2022). However, these findings are based on informant reports for individuals with suspected dementia and may be affected by recollection bias. Praxis was also evaluated by several studies, although only one paper suggested this was sensitive to early decline (Aschenbrenner et al. 2021).

#### 4.6.2 Behavioural and Psychological Symptoms

In total, seven papers included measures of BPSD and personality; however, not all of these included measures of cognition. Common themes included the presence of variable mood and changes in BPSD symptoms, including increased anxious behaviour, irritability, and apathy. Most of the papers reporting these findings compared stable, prodromal, and clinical groups and indicated these changes were present within the prodromal group, suggesting these may be early markers of decline. However, this was not a consistent finding across studies. Firth et al. (2018) concluded that behavioural changes reported by informants occur last when compared to cognitive symptoms such as memory, attention, and EF. Fonseca et al. (2020) also concluded that changes in apathy manifest later than amnestic symptoms. Sleep‐related changes were also indicated to occur in the prodromal stage (Dekker et al. 2021); however, this was the only study to draw conclusions regarding sleep.

#### 4.6.3 Functional and Adaptive Skills

Four papers evaluated functional changes. Changes in ADLs were observed by Wissing et al. (2022) in a sample of individuals with severe/profound intellectual disabilities. They found the prodromal stage was characterised by changes in dressing and eating/drinking skills whereby there was a reduction in how independently individuals could complete these tasks. Similarly, Blok et al. (2017) found that a decline in adaptive functioning was observed for individuals with Down syndrome. Both papers concluded that this decline was not present before changes in memory. Measures pertaining to functional skills and ADLs were informant‐based, which are subject to response bias, including both recall and interpretation of symptoms. It is plausible that respondents' memory of symptom onset was inaccurate, and that symptoms were not conclusively due to dementia.

### 4.7 Determining the Trajectory of Decline

Although all the included studies were interested in changes indicative of early decline, it is unclear what comes first. An added complication in determining the trajectory of decline comes from the methodologies used within studies. For example, four papers did not employ between‐group comparisons, and four papers compared those with clinical dementia to healthy controls, lacking a prodromal comparison. Therefore, it is impossible to infer at what stage symptoms first present, particularly as only three studies used a within‐subjects, longitudinal design. Nevertheless, a number of studies compared those considered healthy, in the prodromal stage, and with a clinical diagnosis. When considering just these papers, a range of conclusions is drawn.

In keeping with research in the general population, five papers concluded that changes in memory are one of the earliest indicators of decline. Several papers suggest that this occurs first, whereas other papers argue memory decline occurs alongside changes in other domains such as attention, language, EF, and motor co‐ordination. Several studies reported changes in EF and behaviour occur in the early stages; however, Blok et al. (2017) found no evidence to suggest these changes occur before memory decline. Firth et al. (2018) suggested a pattern of decline initiated by memory loss, reduced fluency, attention, and motor co‐ordination, followed by changes in planning skills and cognitive flexibility, and finally, behavioural symptoms were observed last. Fonseca et al. (2020) concluded that stable cognition and prodromal dementia are better distinguished by the presence of memory impairments than executive dysfunction, signifying a larger impact of memory decline in the early stage than EF. However, they acknowledged that executive dysfunction underpinned by frontal changes may occur earlier in dementia progression than observed in the general population.

Four papers suggest the presence of non‐cognitive symptoms early in the progression of dementia, including BPSD, verbal fluency, and gait performance; however, they do not directly compare this with the onset of memory decline. The few studies that do draw conclusions regarding the pattern of symptom onset did not use comparator groups and instead based their findings on age, inferring that dementia pathology was likely in the older participants despite the lack of diagnostic support. Although these findings may be due to more than the ageing process, definitive conclusions cannot be drawn.

## 5 Discussion

### 5.1 Summary of Findings

This review aimed to explore early symptoms of dementia in individuals with intellectual disabilities and Down syndrome, focusing on both cognitive and non‐cognitive functions, and considering the trajectory of decline. The findings provide further evidence for the presence of memory decline in the early stages of dementia in these populations and add to a growing body of research which suggests that other abilities are also affected.

Memory, attention, and EF were commonly identified as early symptoms across many of the included studies. In addition, a selection of studies reported that a decline in language, fluency, motor ability, BPSD, and behaviour also occurs early in the disease process. Dementia is often associated with memory impairments, particularly episodic memory, due to medial temporal lobe atrophy (Twamley et al. 2006). Memory decline is often considered to occur first in dementia in both intellectual disabilities, Down syndrome, and the general population (Ball et al. 2008; Deb et al. 2007). Thirteen of the included studies specifically investigated memory, with many studies reporting memory decline as an early symptom of dementia. Hippocampal dysfunction, a structure involved in encoding and memory consolidation, is a likely mechanism (Lott and Dierssen 2010; Rao et al. 2022), and is consistent with storage deficits observed by Benejam et al. (2015). With respect to EF, a large proportion of the included studies emphasised this also being an early feature, consistent with evidence that these difficulties might emerge sooner than previously thought (Deb et al. 2007). Although fewer studies addressed attention, early changes were also observed. It is notable that attention was not a primary focus in most studies despite it being fundamental to many cognitive abilities (Lezak et al. 2012). Nevertheless, the presence of changes in selective attention is supported by a previous study whereby a progressive pattern of decline in selective attention was observed in early‐stage AD (Krinsky‐McHale et al. 2008). Participants performed significantly worse than healthy peers on a cancellation task and demonstrated greater variability across trials. This study also used a cancellation task to measure selective attention; therefore, these findings may not be generalisable beyond this measure.

Regarding non‐cognitive domains, early changes in mobility and BPSD may signal dementia, though findings are mixed. Ball et al. (2006) concluded that personality and behavioural changes are prominent features of AD even in the absence of memory decline. Conversely, Adams and Oliver (2010) observed that behavioural changes in Down syndrome only occur in those also demonstrating cognitive decline. However, it is plausible that changes in non‐cognitive abilities are underpinned by changes in cognition, rather than being an independent feature. Hughes et al. (2020) identified a relationship between EF and mobility in older adults without intellectual disabilities, suggesting that executive dysfunction might be linked with greater decline in mobility. Despite this, mobility decline may be more readily observed in the prodromal stage than memory or EF, particularly in those with intellectual disabilities.

Whilst this review enhances understanding of early dementia symptoms, there remains no clarity on the earliest affected domain, with studies drawing conflicting conclusions. Better understanding and improved detection of early symptoms is essential to provide the best support for individuals (Llewellyn 2011). However, changes to assessment persist, including individual heterogeneity, pre‐existing cognitive deficits, and a lack of standardised approaches (Ballard et al. 2016). The inclusion of domains beyond just memory may be a helpful next step in improving dementia diagnosis in this population.

### 5.2 Clinical Implications

Although previous research suggests dementia in individuals with intellectual disabilities and Down syndrome progresses systematically rather than manifesting globally (Devenny et al. 2000), this review suggests that early routine assessments should include measures of memory, EF, language, attention, BPSD, and motor/mobility. These findings have implications for not only the process of dementia assessments but also care‐planning and the provision of care.

In line with British Psychological Society and the Royal College of Psychiatry guidance (2015); British Psychological Society and the Royal College of Psychiatry guidance (2025) on dementia assessment in this population, involving professionals and family members in assessments is essential. They have a comprehensive understanding of the individual with suspected dementia and can provide unique insights into their functioning over time, often raising concerns first. As such, it is important that all key stakeholders are aware of cognitive and non‐cognitive changes that may indicate the presence of dementia in order to facilitate earlier assessments and lead to a timelier diagnosis. Subsequently, this can aid more effective post‐diagnostic support. With a clearer definition of prodromal dementia in adults with intellectual disabilities and Down syndrome, this opens up scope to develop measures that are sensitive to this specific stage of decline (Krinsky‐McHale et al. 2020).

In light of these findings, policies need to evolve (Burke et al. 2018). If non‐cognitive symptoms do emerge prior to cognitive decline, guidance should be reviewed to encourage assessment of these domains and ensure baseline assessments are broad in nature (Prasher et al. 2015). Although informant‐based assessments often incorporate social scales including measures of practical skills, mood, activity, interest, and behavioural disturbance, direct measures of these areas are less well utilised. Historically, many tools used to assess dementia in adults with intellectual disabilities have relied heavily on memory measures, due to the well‐established link between early‐onset Alzheimer's Disease and Down syndrome. The development of measures such as the CAMDEX‐DS and its adoption by NHS services enable the evaluation of adaptive skills in addition to a wide range of cognitive domains. It is important that a well‐validated measure is established, encompassing a broader range of domains, including but not limited to EF, language, and mobility.

As life expectancy increases in this population, early detection should be prioritised to support the wellbeing and resource allocation. Policies should stress the need for routine baseline assessments when individuals are functioning well, as often there are delays in diagnosis due to the need for repeated assessment. Additionally, raising awareness of modifiable risk factors of dementia, including social isolation and a sedentary lifestyle, should be promoted (Takenoshita et al. 2023), should be emphasised.

### 5.3 Methodological Considerations

Several methodological limitations need consideration when attempting to explore these early symptoms. Firstly, although many studies had large samples, most focused on individuals with Down syndrome, rather than the broader intellectual disability population. As such, although the original focus of this review was to explore the presentation of dementia in individuals with intellectual disabilities generally, this paper primarily reflects early markers of dementia in Down syndrome, given the sample bias of the included papers. Additionally, where dementia sub‐groups were utilised, participant numbers were often small, limiting statistical power and generalizability. Additionally, group classifications varied across studies. Four studies lacked a comparator group, three studies compared healthy controls and those with dementia, one study compared healthy controls with possible dementia, and ten studies used multiple groups (healthy, prodromal, clinical). The absence of groups, particularly the prodromal group, makes it difficult to isolate symptoms that develop prior to a clinical diagnosis. Whilst some studies reported memory, language, and praxis as being sensitive to early decline (Aschenbrenner et al. 2021), clear conclusions cannot be drawn. Only three studies were longitudinal, offering the advantage of tracking change over time, rather than comparing heterogeneous groups as in cross‐sectional studies (Wang and Cheng 2020). The longitudinal studies could also review scores retrospectively, in light of later diagnoses.

A further limitation relates to the conceptualisation of cognitive functions. For example, a proportion of studies explored verbal fluency as a separate construct; however, other studies combined it with language or EF. This lack of consistency complicates interpretation. Additionally, there are issues related to the measures used and their lack of validity in the intellectual disability population. For example, Martin et al. (2000) found that although individuals with intellectual disabilities could complete the Rivermead Behavioural Memory Test, its appropriateness for determining memory impairments is unclear. Methodological differences may explain observed group differences more than true variation.

The impact of normal ageing and premorbid abilities cannot be discounted. In previous research exploring assessment tools for dementia in intellectual disability, individuals with severe/profound intellectual disabilities have been excluded (Crayton et al. 1998). Therefore, it is difficult to determine trajectories across the intellectual disability population and whether variation in symptomology is based on pre‐existing cognitive abilities. Aschenbrenner et al. (2021) highlighted that those with severe intellectual disability have lower baseline abilities, which may limit measurable decline. Similarly, to previous reviews, many included studies only provided details of clinical characteristics rather than comparisons between domains and the course of decline (Lautarescu et al. 2017). Subsequently, it remains unclear how symptoms develop over time in this population.

Finally, adopting the approach of combining intellectual disabilities and Down syndrome to explore dementia markers could be problematic. Whilst Alzheimer's disease is the most common dementia in both groups, other types (e.g., vascular, frontotemporal, Lewy body) are more prevalent in non‐Down syndrome populations (Strydom et al. 2007, 2010). Further, it is possible that the presentation of these dementia sub‐types involves different prodromal features which may be overlooked by looking collectively at intellectual disabilities and Down syndrome. Despite this, population‐based comparisons can still inform clinical practise; however, the lack of studies specifically investigating the prodromal stage of dementia in individuals with intellectual disability without Down syndrome limits such comparisons.

### 5.4 Strengths and Limitations of the Review

A strength of this review is its attempt to combine research across multiple domains, rather than looking at abilities in isolation. The scope of this review was initially broad, to reduce the likelihood of not capturing relevant articles. However, a limitation is that the final iteration of studies used samples primarily of individuals with Down syndrome; therefore, these findings cannot be applied to the intellectual disability population. It is likely that this is more a reflection of the evidence base lacking studies exploring dementia in intellectual disabilities, rather than the review methodology. Ethical issues surrounding consent and capacity may in part explain this, creating barriers to research and reducing the representativeness of samples, as those who lack capacity to consent are often excluded (Goldsmith and Skirton 2015). This often relates to researcher confidence in consent and capacity processes, lack of clear guidance from ethics committees, and inaccessible research designs (Bishop et al. 2023). Individuals who lack capacity, or whose capacity may fluctuate, should be provided with opportunities to partake in research, particularly regarding aspects that affect their lives. However, they often require support to exercise their legal rights to engage in research. Despite this, further efforts should be made to increase the inclusivity of research. Additionally, memory was the most studied aspect of cognition, which could bias the conclusions, suggesting it is more common as an early symptom than it truly is.

Another limitation is the lack of consistency in how studies categorised dementia groups, and the inclusion of studies where longitudinal or between‐group comparisons were not adopted. This resulted in difficulties synthesising and comparing data across studies. Additionally, the vast heterogeneity of measures used further complicates this. A smaller, more homogeneous selection of measures would be of benefit in synthesising the findings and in providing recommendations of suitable measures for use in detecting dementia in this population. Previous reviews have encountered similar challenges, with minimal overlap in measures used and suggest this is a reflection of the lack of standardized measures available (Paiva et al. 2020). The quality assessment indicated the majority of studies were of moderately good quality. Objectives and samples were sufficiently described; however, methods of comparison and the control of confounding variables were lacking, which are important factors for determining differences in prodromal dementia. Although the assessment tool used encouraged reviewers to consider sources of bias and their impact, interpretation was largely subjective.

### 5.5 Recommendations for Future Research

Future research should seek to further verify the trajectory of decline in dementia for this population, focusing on prodromal features. Much of the evidence base continues to utilise samples consisting predominantly of individuals with Down syndrome. As a result, our understanding of early dementia in intellectual disabilities across the range of cognitive abilities remains limited. This review has established further evidence for a differing picture of dementia progression than previously assumed. It is imperative that additional, methodologically sound research is conducted to provide clarity regarding early symptoms and progression, with clear criteria for classifying prodromal dementia and adopting validated measures for this population. Research should seek to use a more focused range of measures, encompassing important domains including EF, attention, language, mobility, and BPSD. Furthermore, much of the current research has focused on samples in Western populations. Future research should consider dementia in different ethnicities, ensuring the individuals with intellectual disabilities and Down syndrome from ethnic minorities are also represented and their needs considered.

## 6 Conclusions

In conclusion, the narrative synthesis presented provides insight into the early symptomology of dementia in intellectual disabilities and Down syndrome, pulling together a far wider range of domains than previous reviews. Memory, EF, language, attention, behaviour, and motor skills/mobility have been proposed as early indicators of prodromal dementia in this population and provide support for the need for broader assessments when concerns regarding dementia are raised. There remains a lack of agreement as to the onset of these symptoms chronologically. Further methodologically sound studies are needed to explore this.

## Author Contributions

**Jade Dunning:** conceptualisation, methodology, data analysis, writing – original draft and reviewed manuscript. **Melanie Hodgkinson:** conceptualisation, methodology, writing – reviewing and editing, supervision. **Mark Rose:** conceptualisation, writing – reviewing and editing. **Warren Dunger:** conceptualisation, methodology, writing – reviewing and editing, supervision.

## Disclosure

The research is the product of a Doctorate in Clinical Psychology thesis supported by the University of Southampton.

## Ethics Statement

The authors have nothing to report.

## Consent

The authors have nothing to report.

## Conflicts of Interest

The authors declare no conflicts of interest.

## Supporting information


**Table S1:** Search terms and databases.

**Table S2:** Quality assessment for the included papers using the QualSyst tool.

**Table S3:** Measures used in included studies and the frequency of use.

## Data Availability

The data that support the findings of this study are available from the corresponding author upon reasonable request.
