# Supplementary material for: Early Markers for Dementia in the Intellectual Disability Population: A Systematic Literature Review
Source: J Appl Res Intellect Disabil. 2025 Oct 23;38(5):e70144. doi: 10.1111/jar.70144 (PMC12550045; doi:10.1111/jar.70144)
Supplement: Supplementary file 1 — Table S1: Search terms and databases. [file JAR-38-e70144-s001.docx]

**Supplementary Table 1**

*Search Terms and Databases*

| Database |  | Search Terms | | | | |
| --- | --- | --- | --- | --- | --- | --- |
| PsychInfo |  | Intellectual Disability Terms  (intellectual* OR learning* OR mental* OR developmental*)N1(disab* OR retard* OR handicap))  OR  “Down* Syndrome*” OR trisomy* 21 | AND | Dementia Terms  Dementi* OR Alzheimer* | AND | Symptoms Terms  Symptom* OR marker* OR sign* OR feature* OR presentation* OR change* |
| PubMed |  | (intellectual*) OR (learning*) OR (mental*) OR (developmental*)  AND  (disab*) OR (retard*) OR (handicap*)  OR  (“Down* syndrome”) OR (trisomy* 21) | AND | (dementia*) OR (Alzheimer*) | AND | Symptom* OR marker* OR sign* OR feature* OR presentation* OR change* |
| Web of Science |  | (intellectual*) OR (learning*) OR (mental*) OR (developmental*)  AND  (disab*) OR (retard*) OR (handicap*)  OR  ("Down* syndrome") OR (trisom* 21) | AND | (dementia*) OR (Alzheimer*) | AND | (symptom* OR marker* OR sign* OR feature* OR presentation* OR change*) |
