# Supplementary material for: Early Markers for Dementia in the Intellectual Disability Population: A Systematic Literature Review
Source: J Appl Res Intellect Disabil. 2025 Oct 23;38(5):e70144. doi: 10.1111/jar.70144 (PMC12550045; doi:10.1111/jar.70144)
Supplement: Supplementary file 2 — Table S2: Quality assessment for the included papers using the QualSyst tool. [file JAR-38-e70144-s003.docx]

**Supplementary Table 2**

*Quality Assessment for the Included Papers using the QualSyst Tool*

| Author and Year | Checklist Item | | | | | | | | | | | | | | Summary score (JD) | Second rater summary score (AM) | Inter-rater agreement |
| --- | --- | --- | --- | --- | --- | --- | --- | --- | --- | --- | --- | --- | --- | --- | --- | --- | --- |
|  | 1 | 2 | 3 | 4 | 5 | 6 | 7 | 8 | 9 | 10 | 11 | 12 | 13 | 14 |  |  |  |
| Arvio & Bjelogrlic-Laakso (2021) | 1 | 2 | 2 | 1 | n/a | n/a | n/a | 1 | 2 | 1 | 1 | 0 | 1 | 2 | 0.64 | - | - |
| Aschenbrenner et al. (2021) | 2 | 1 | 2 | 2 | n/a | n/a | n/a | 2 | 2 | 2 | 1 | 0 | 2 | 2 | 0.77 | 0.82 | 86% |
| Benejam et al. (2015) | 2 | 2 | 2 | 1 | n/a | n/a | n/a | 2 | 1 | 2 | 2 | 1 | 2 | 2 | 0.86 | 0.82 | 71% |
| Benejam et al. (2020) | 2 | 2 | 2 | 2 | n/a | n/a | n/a | 1 | 2 | 1 | 2 | 1 | 2 | 2 | 0.86 | - | - |
| Blok et al. (2017 | 2 | 1 | 1 | 1 | n/a | n/a | n/a | 2 | 1 | 2 | 2 | 1 | 2 | 2 | 0.77 | - | - |
| Conceição et al. (2023) | 2 | 2 | 2 | 2 | n/a | n/a | n/a | 1 | 1 | 2 | 1 | 1 | 2 | 2 | 0.82 | 0.83 | 86% |
| Dekker et al. (2021) | 2 | 1 | 1 | 2 | n/a | n/a | n/a | 2 | 2 | 2 | 2 | 2 | 2 | 1 | 0.86 | - | - |
| Firth et al. (2018) | 2 | 2 | 1 | 2 | n/a | n/a | n/a | 2 | 2 | 1 | 0 | 1 | 1 | 1 | 0.68 | - | - |
| Fonseca et al. (2019) | 2 | 2 | 1 | 2 | n/a | n/a | n/a | 1 | 2 | 2 | 1 | 0 | 2 | 1 | 0.73 | - | - |
| Garcia-Alba et al. (2019) | 2 | 2 | 1 | 1 | n/a | n/a | n/a | 1 | 1 | 2 | 1 | 1 | 2 | 2 | 0.73 | - | - |
| Hartley et al. (2020) | 2 | 2 | 2 | 2 | n/a | n/a | n/a | 1 | 2 | 1 | 1 | 0 | 2 | 1 | 0.73 | - | - |
| Hom et al. (2021) | 2 | 2 | 1 | 2 | n/a | n/a | n/a | 2 | 2 | 2 | 1 | 1 | 2 | 2 | 0.86 | - | - |
| Mgaieth et al. (2023) | 2 | 1 | 1 | 2 | n/a | n/a | n/a | 2 | 2 | 1 | 1 | 2 | 2 | 2 | 0.82 | - | - |
| Pulsifer et al. (2020) | 2 | 2 | 2 | 2 | n/a | n/a | n/a | 1 | 2 | 1 | 2 | 1 | 2 | 2 | 0.86 | - | - |
| Startin et al. (2019) | 1 | 2 | 1 | 2 | n/a | n/a | n/a | 1 | 2 | 2 | 1 | 2 | 2 | 2 | 0.82 | - | - |
| Van Pelt et al. (2020) | 2 | 1 | 1 | 2 | n/a | n/a | n/a | 1 | 2 | 2 | 0 | 2 | 2 | 2 | 0.77 | - | - |
| Wissing et al. (2022) |  |  |  |  |  |  |  |  |  |  |  |  |  |  |  |  |  |
| Quantitative checklist: | 1 | 2 | 2 | 1 | n/a | n/a | n/a | 1 | n/a | 2 | 0 | n/a | 2 | 2 | 0.72 | 0.77 | 71% |
| Qualitative checklist: | 1 | 2 | 2 | 1 | 2 | 2 | 2 | 2 | 2 | 0 | - | - | - | - | 0.80 | 0.90 | 80% |
| Wissing et al. (2023) | 2 | 1 | 2 | 2 | n/a | n/a | n/a | 1 | 1 | 2 | 1 | 1 | 2 | 2 | 0.77 | - | - |

*Note.* Responses scored as 0 = No, 1 = Partial, 2 = Yes, n/a = Not Applicable. Cell shading represents responses to statements: green = ‘Yes’, orange = ‘Partial’, red = ‘No’.
