# Supplementary material for: Early Markers for Dementia in the Intellectual Disability Population: A Systematic Literature Review
Source: J Appl Res Intellect Disabil. 2025 Oct 23;38(5):e70144. doi: 10.1111/jar.70144 (PMC12550045; doi:10.1111/jar.70144)
Supplement: Supplementary file 3 — Table S3: Measures used in included studies and the frequency of use. [file JAR-38-e70144-s002.docx]

**Supplementary Table 3**

*Measures Used in Included Studies and the Frequency of Use*

| Measure | Frequency |
| --- | --- |
| Direct Tools: |  |
| Scales and Questionnaires: |  |
| British Present Psychiatric State-Learning Disabilities Scale (PPS-LD) | 1 |
|  |  |
| Batteries: |  |
| NEPSY 2^nd^ edition: A Developmental Neuropsychological Assessment (NEPSY-II) | 1 |
| Cambridge Cognitive Examination for Older Adults with Down’s   Syndrome (CAMCOG-DS) | 4 |
| Cambridge Examination for Mental Disorders of Older People with Down’s Syndrome and Others with Intellectual Disabilities (CAMDEX-DS) | 4 |
| Severe Impairment Battery (SIB) | 2 |
| Kaufman Brief Intelligence Test (KBIT) | 2 |
| Kaufman Brief Intelligence Test 2^nd^ edition (KBIT-2) | 4 |
| The Rapid Assessment of Developmental Disabilities – Second Edition (RADD-2) | 1 |
|  |  |
| Tests: |  |
| Boston Naming Test (BNT) | 3 |
| Peabody Picture Vocabulary Test-4^th^ Edition (PPVT) | 1 |
| Rivermead Behavioural Memory Test (RBMT) | 1 |
| Brief Praxis Test (BPT) | 2 |
| Purdue Pegboard Test (PPT) | 3 |
| Timed Up and Go (TUG) | 2 |
| Modified Cued Recall Test (mCRT) | 2 |
| Visual Association Test (VAT) | 1 |
| Performance-Oriented Mobility Assessment (POMA) | 1 |
| The Beery Buktenica Developmental Test of Visual-Motor Integration | 1 |
| Tinetti Balance and Gait Assessment Tool | 1 |
|  |  |
| Tasks: |  |
| Verbal Fluency | 3 |
| McCarthy Verbal Fluency | 1 |
| Semantic Verbal Fluency | 1 |
| Spatial Reversal, taken from Cambridge Executive Functioning Assessment (CEFA) | 1 |
| Scrambled Boxes, taken from CEFA | 1 |
| Cats & Dogs, taken from CEFA | 2 |
| Tower of London, taken from CEFA | 2 |
| Finger-nose pointing | 3 |
| Paired Associates Learning (PAL), taken from the Cambridge Neuropsychological Test Automated Battery (CANTAB) | 3 |
| Intra/extra Dimensional Set Shift (IED) task, taken from CANTAB | 3 |
| Simple Reaction Time (SRT) task, taken from CANTAB | 4 |
| Digit Span | 1 |
| Corsi Block-Tapping Test (CBT) Forward | 1 |
| Corsi Block-Tapping Test (CBT) Backward | 1 |
| Object Memory | 2 |
| Cancellation Task | 1 |
| 4-Meter Walk | 1 |
| Fuld Object Memory Test | 1 |
| Orientation | 2 |
| Car and Motorbike Score, taken from NEPSY-II | 1 |
| Cued Recall Test, taken from NEPSY-II | 3 |
| Visuomotor Perception, taken from NEPSY-II | 1 |
| Visuospatial Precision, taken from NEPSY-II | 1 |
| Temporal Orientation Test (TO) | 1 |
| Delayed Visual Memory (DVM) | 1 |
| Auditory Delayed Verbal Memory (ADVM) | 1 |
| Cat and Dog Modified Stroop Task | 1 |
| Block Design | 3 |
| GAITRite^TM^ | 1 |
| Riddles, taken from the KBIT | 1 |
| Visuo-motor Integration (VMI) | 1 |
| Story Recall | 1 |
| Ideomotor Praxis | 1 |
| Adapted version of Tower of London | 1 |
| Adapted Category Fluency Test | 1 |
| Down Syndrome Mental Status Examination (DSMSE) | 1 |
| Modified MMSE | 1 |
|  |  |
| Informant-based Questionnaires: |  |
| Dementia Questionnaire for People with Learning Disabilities (DLD) *Previously called* Dementia Questionnaire for Persons with Mental (DMR) | 6 |
| Temperament Scale for People with Intellectual Disability (TVZ) | 1 |
| Social Disability Scale for the Mentally Retarded (SRZ/SRZ-i) | 1 |
| Short Adaptive Behavior Scale (Short ABS) | 2 |
| Observer Memory Questionnaire (OMQ) | 2 |
| Vineland Adapted Behavior Scale, taken from Vineland 2^nd^ edition (Vineland-II) | 1 |
| Behavior Rating Inventory of Executive Function – Adult version (BRIEF-A) | 1 |
| Working Memory (WM), taken from Behavior Rating Inventory of Executive Function-Parents (BRIEF-P) | 1 |
| Communication Domain, taken from Vineland-3 | 1 |
| Behavioral and Psychological Symptoms of Dementia in Down Syndrome II (BPSD-DS II) | 1 |
|  | Total = 66 |
